# Hierarchical Event-Control and Subjective Experience of Agency

**DOI:** 10.3389/fpsyg.2012.00410

**Published:** 2012-10-17

**Authors:** Devpriya Kumar, Narayanan Srinivasan

**Affiliations:** ^1^Centre of Behavioural and Cognitive Sciences, University of AllahabadAllahabad, India

Clark (in press) has argued that our brain uses hierarchical predictive coding and the use of this concept would enable us to understand the mental life of the organism. We agree that hierarchical predictive coding provides a good framework for developing further theories and models for different aspects of brain functioning including, perception, action, attention, and experience.

An important but least understood aspect of human cognition is the “I” as an experiencer. A person perceives one's self as an agent or controller of actions through one's interactions with the environment. This influences the perception of the event itself. Events that are felt to be caused by self are perceived differently from other events. For example, subjective experience of flash lag effect reduces when the subject plays an active part in the event (Ichikawa and Masakura, 2010). Intentional binding between an action and a tone has been reported to become stronger when subjects experience greater control over the action (Moore et al., 2009).

Clark has pointed out that models of predictive coding may shed some light on aspects of subjective experience of agency (possibly self) and enable us to computationally model these experiences. A prediction based model that deals with self is the Bayesian version of the comparator model (Fletcher and Frith, 2009). In this model, agency is based on the successful prediction of the organisms’ sensory consequences of action performed by the organism. This model has also been used as a major component in the predictive coding based model of conscious presence (Seth et al., 2012). While Clark in his paper has emphasized on the formation of subjective experiences, it is not immediately clear how self can be understood in the context of hierarchical predictive coding.

One possible way to understand self and agency is through the notion of event-control (Jordan, 2003) which argues that self gets attached to a particular level of event-control at a given time. Using the example of driving, for an inexperienced driver, self would get attached to the proximal level of control related to hand or leg movements and the clutch, brake, and gear. However, an experienced driver for whom driving is fairly automatic self would get attached to the successful achievement of the goal, that is, driving from one place to another.

Event-control approach proposes the notion of hierarchically arranged control loops (Jordan, 2003) that are possibly nested (Feinberg, 2011), with the distal goal at the top-level and the proximal sensory-motor loop at the lowest level. There is both a feed-forward and a feed-back information transfer between different levels in the hierarchy. Each level constrains the activity of the lower levels by fixing certain parameters (the lower level control loops function under the constraints provided by the higher control loop). In return what is fed back to the higher level is the amount of control that the lower level could achieve given the constraints. Using this information, the constraints are modified so as to achieve efficient control by the lower levels.

In this approach, the subjective sense of self is not attached to a particular loop (Frith, 2005; Seth et al., 2012). It rather varies dynamically as a function of the successful control achieved at a particular level. The sense of self is conceptualized as fluid or plastic with self attached to the highest level in which control is successfully exercised at a particular level in the hierarchy. The level at which “self” gets attached could change as an organism continues to interact with the environment in the context of an intentional goal, moving across different levels in the hierarchy at different times.

Empirical data from studies exploring the link between sense of self and event-control (Jordan, 2003; Kumar and Srinivasan, under review) suggest that participant's sense of self does vary with the amount of control that they can exercise. We have studied the emergence of self as a function of different levels in a event-control hierarchy using a multi-agent paradigm (Kumar and Srinivasan, under review). When control is manipulated at multiple levels, self gets attached to the higher/more distal level at which control can be successfully exercised. When the exercise of control is poor at a higher level, the sense of self attaches to the lower (more proximal) level of event-control. The results also show that when people misidentify as some other agent pursuing a common goal, misidentification is more when the other agent affords a greater experienced control (again in a hierarchical fashion). The results based on event-control approach is consistent with the studies that have shown a link between the feeling of control and the subjective experience of agency (Frith, 2005; Moore et al., 2009; Dewey et al., 2010; Desantis et al., 2011).

The event-control approach goes beyond other hierarchical predictive coding models of subjective experience (Frith, 2005; Seth et al., 2012), not conceptualizing self in terms of what is happening at the pre-fixed top-level module in the hierarchy. It visualizes a dynamic fluid self that depends on control exercised at a particular level and changes as a function of an organism's interactions with the environment. This enables us to understand changes in self and identity as a function of interaction between organism and the environment (as more situated and embodied) rather than simply focusing on the organism *per se*. In addition, self is neither visualized as a cause of decisions made by the organism nor as epiphenomenal but rather in terms of experience that emerges out of regularities in perception-action events.

To conclude, we broadly agree with the notion of hierarchical predictive coding and extend it to explain the emergence of self and the experience of agency by naturalizing them as the level of control achieved at a given level in the hierarchy involving perceptual-action events. The use of hierarchical control systems would enable us to study our mental life and better understand the epistemic concepts of intentionality, consciousness, and self.
